# Up-Regulation of TIMP-1 by Genipin Inhibits MMP-2 Activities and Suppresses the Metastatic Potential of Human Hepatocellular Carcinoma

**DOI:** 10.1371/journal.pone.0046318

**Published:** 2012-09-28

**Authors:** Ning Wang, Meifen Zhu, Sai-Wah Tsao, Kwan Man, Zhangjin Zhang, Yibin Feng

**Affiliations:** 1 School of Chinese Medicine, Li Ka Shing Faculty of Medicine, The University of Hong Kong, Hong Kong, People’s Republic of China; 2 Department of Anatomy, Li Ka Shing Faculty of Medicine, The University of Hong Kong, Hong Kong, People’s Republic of China; 3 Department of Surgery, Li Ka Shing Faculty of Medicine, The University of Hong Kong, Hong Kong, People’s Republic of China; Duke University Medical Center, United States of America

## Abstract

**Aim of the Study:**

Hepatocellular carcinoma is one of the most malignant human cancers with high metastatic potential. The aim of this study is to investigate the anti-metastatic effect of genipin and its underlying mechanism.

**Experimental Approach:**

The anti-metastatic potential of genipin was evaluated by both cell and animal model. Wound healing and invasion chamber assays were introduced to examine the anti-migration and anti-invasion action of genipin in human hepatocellular carcinoma cell HepG2 and MHCC97L; orthotopical implantation model was used for in vivo evaluation. Gelatin Zymography, Immunoblotting, quantitative real-time polymerase chain reaction and ELISA assays were used to study the mechanisms underlying genipin’s anti-metastatic effect.

**Key Results:**

Genipin suppresses the motility and invasiveness of HepG2 and MHCC97L at non-toxic doses, which may be correlated to the inhibition of genipin on MMP-2 activities in the cells. No significant reduced expression of MMP-2 was observed either at mRNA or at protein level. Furthermore, genipin could specifically up-regulate the expression of TIMP-1, the endogenous inhibitor of MMP-2 activities. Silencing of TIMP-1 by RNA interference abolishes genipin’s anti-metastaic effect. Activation of p38 MAPK signaling was observed in genipin-treated cells, which is responsible for the TIMP-1 overexpression and MMP-2 inhibition. Presence of SB202190, the p38 MAPK inhibitor, attenuates the anti-metastatic potential of genipin in hepatocellular carcinoma. Orthotopical implantation model showed that genipin could suppress the intrahepatic metastatic as well as tumor expansion in liver without exhibiting potent toxicity.

**Conclusion:**

Our findings demonstrated the potential of genipin in suppressing hepatocellular carcinoma metastasis, and p38/TIMP-1/MMP-2 pathway may be involved as the key mechanism of its anti-metastasis effect.

## Introduction

Hepatocellular carcinoma (HCC) is one of the most malignant human cancers all over the world. As the primary cancer of the liver, HCC accounts for over 85% of the liver cancer cases [1]. Hepatocellular carcinoma was newly diagnosed in more than half of million people in the world annually [2], and has become one of the most leading causes of death worldwide. It is the fifth common malignant tumor in men while seventh common in women [1], and it is the third common causes of cancer mortality all over the world with the incidence rates are increasing every year [3]. Although currently different therapeutic strategies have developed, the prognosis of HCC remains poor due to the high reoccurrence rate and metastatic effect of HCC cells [4]. Incidence of intrahepatic metastatic is high in hepatocellular carcinoma with infiltrate growth pattern according to clinicopathologic study [5]. However, there’s no effective chemotherapeutic agent which could prevent metastasis in hepatocellular carcinoma patients.

Genipin, the metabolite of geniposide, is a natural product present in fruit of *Gardenia jasminoides*. Previous study shows that geniposide is absorbed and transformed to genipin in the bowel, indicating that genipin may be the major form of geniposide in blood [6]. Genipin has been report to have anti-inflammatory [7], anti-oxidative [8], anti-thrombotic [9] and neuroprotective activities [10]. Some recent studies also present the anti-tumor effect of genipin in some human cancer cells including cervical cancer cells Hela, hepatoma cells Hep3B and prostate cancer cells PC3 by inducing cell apoptosis [11]–[13]. However, there’s no study reporting the anti-metastasis effect of genipin and its underlying mechanism in human hepatocellular carcinoma.

In the present study, we investigated the anti-invasive effect of genipin on human hepatocellular carcinoma. We found that genipin exhibits no significant cytotoxicity to human hepatocellular carcinoma cells HepG2 and MHCC97L, however, genipin could remarkably suppress the migration and invasion of the HCC cells. Genipin presents inhibitory effect on the MMP-2 activities, which is responsible for the invasiveness of cancer cells. Genipin has no direct inhibitory effect on the enzyme activities of MMP-2 in vitro, instead, up-regulation of the MMP-2 inhibitor TIMP-1 by genipin in HCC cells may contribute to its inhibition on MMP-2 enzyme activities. In addition, activation of p38 MAPK signaling by genipin may be responsible for its anti-invasive effect in HCC. Animal study confirms that genipin could suppress the invasion of HCC cells. Our study sheds light on the potential inhibition of genipin on hepatocellular carcinoma metastasis and some novel mechanisms may be involved.

## Results

### Genipin Inhibits the HCC Cells Migration and Invasion in Non-toxic Manner

**Figure 1:**
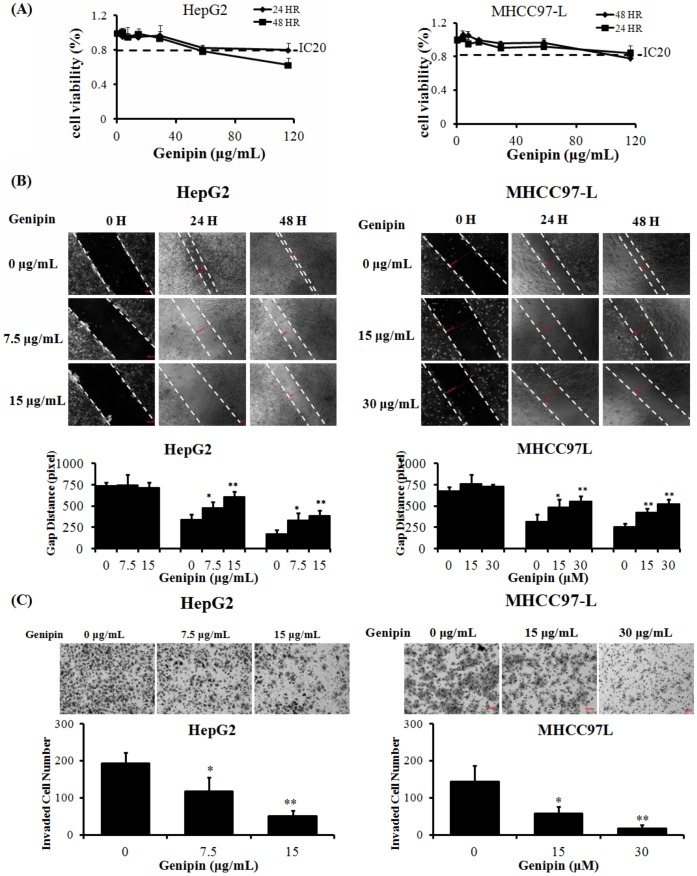
Genipin suppresses hepatocellular carcinoma cell migration and invasion at non-toxic doses. **A** shows that genipin exhibits no potent cytotoxicity to human heaptocellular carcinoma cells HepG2 and MHCC97L. Cells were seeded in 96-well culture plate and treated with genipin for 24 h or 48 h. Cell viability was determined by MTT assay. **B** shows that genipin remarkably reduce HepG2 and MHCC97L cell motility in dose-dependent manner. Cells were seeded in 12-well culture plate with 100% confluence. Wound healing assay was introduced to determine the cell motility in the presence of genipin. The distance between the gap was quantified and the results were shown. *p<0.05, **p<0.01 when compared with control; **C** shows that genipin could inhibit cell invasion through extracellular matrix. Cells were seeded in the chamber of transwells coated with Matrigel matrix. The invasiveness of cells was determined by counted cells passing through Matrigel matrix to the basal side of transwells. *p<0.05, **p<0.01 when compare with vehicle group.

In our study, we found that genipin exerts no significant cytotoxicity to human hepatocellular carcinoma cells HepG2 and MHCC97L for either 24 hr or 48 hr treatment. Cells with genipin exposure present no potent reduction in viability at the dose of not more than 60 µg/mL in HepG2 cells and at not more than 120 µg/mL in MHCC97L cells (Fig. 1A). This indicates that genipin has no inhibitory action on cell proliferation or cell survival. Interestingly, we found that genipin exerts remarkable inhibitory action on cell migration on 1-D surface as well as cell invasion through extracellular matrix materials at the dose far lower than its cytotoxicity. 7.5 µg/mL and 15 µg/mL treatment to HepG2 cells or 15 µg/mL and 30 µg/mL treatment to MHCC97L cells could significantly reduce cancer cell migration and invasion, as indicated by wound healing assay (Fig. 1B) and Matrigel Matrix invasion assay (Fig. 1C). Our result indicates that genipin is a potent inhibitor for hepatocellular carcinoma cell migration and invasion in cytotoxic-independent manner.

### Genipin Down-regulates MMP-2 Activity

**Figure 2:**
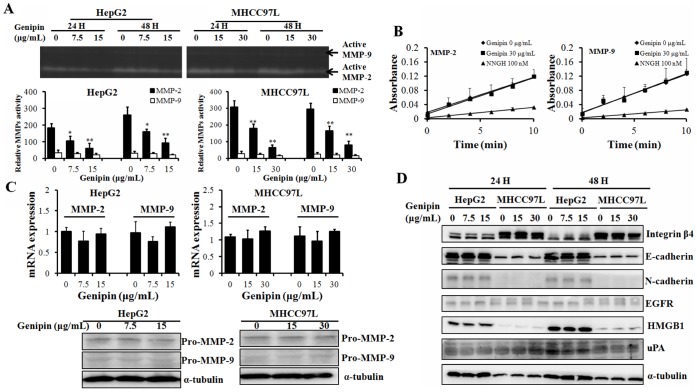
Genipin suppresses MMP-2 activities without inhibiting their expression in hepatocellular carcinoma cells. **A** shows that genipin could inhibit MMP-2 activities in dose- and time-dependent manner in HepG2 and MHCC97L cells. Cells were treated with genipin for 24 or 48H then medium was collected. The MMP-2 and MMP-9 activities were examined by gelatin zymography. Intensity of the band was quantified. *p<0.05; **p<0.01 when compared with control; **B** shows that genipin has no effect on in vitro recombinant MMP-2 and MMP-9 enzyme activity. The MMP-2 and MMP-9 enzymatic dynamics in the presence of 160 µM of genipin were determined as described. No significant change on the dynamic slope could be observed upon genipin treagtment. **C** shows that genipin does not inhibit the mRNA transcript and protein expression. Cells were treated with genipin for either 24 H and then total RNA and protein were collected as described. The mRNA transcript expression was examined by quantitative real-time PCR while protein expression was determined by immunoblotting. **D** shows that no significant alternation on the other major signaling transductions of cell migration and invasion in genipin-treated HCC cells. HepG2 and MHCC97L cells were treated with genipin for 24 or 48H and protein was collected for immunoblotting analysis.

The MMPs activities were detected with Gelatin Zymography assay and inhibition of genipin on MMP-2/9 activity was observed in HepG2 and MHCC97L cells with treatment of genipin (Fig. 2A). The expression and activity of MMP-9 were rather low in HCC cells and although suppression of MMP-9 activity could be found in genipin-treated cells, there’s no statically significant between treated cells and non-treated cells. In contrast, the basal levels of MMP-2 expression and activity in HCC cell were much greater than MMP-9, and genipin exhibit statistically significant inhibition on MMP-2 activity. These observations indicate that the anti-migration action of genipin may major attribute to MMP-2 activity suppression. Genipin shows no inhibitory effect on the activities of human recombinant MMP-2/9. The enzymatic reactions of MMP-2/9 could not be directly blocked or postponed by genipin treatment even at rather high dose (Fig. 2B). To determine whether the reduced activity of MMP-2 is attributable to the suppression of protein expression in hepatocellular carcinoma cells, we detected the mRNA transcripts and protein levels of MMP-2/9 in HepG2 and MHCC97L cells with semi-quantitative PCR and western blot, respectively. Genipin has no significant effect on the expression of MMP-2/9 at either mRNA transcripts or protein level (Fig. 2C). In addition, genipin exhibits no remarkable effect on other migration and invasion related conventional signaling in both HepG2 and MHCC97L cells (Fig. 2D). This may indicate that genipin could downregulate the MMP-2 activity by inhibiting its cleavage activation but not suppressing its expression in hepatocellular carcinoma cells.

### Increased Expression of TIMP-1 by Genipin Contributes to its Inhibitory Effect on MMP-2 Activity in HCC Cells

**Figure 3:**
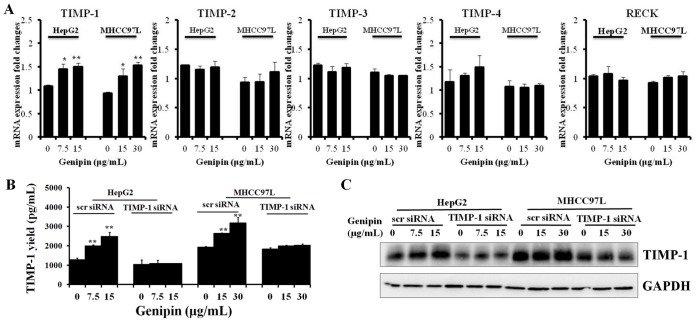
Genipin treatment up-regulates the expression of TIMP-1 in HCC cells. **A** shows the quantitative real-time PCR screening of endogenous MMP-2/9 inhibitors. Cells were treated with genipin for 24H and total RNA was collected for anlaysis. The result shows that genipin particularly up-regulate TIMP-1 mRNA transcription but has no effect on TIMP-2, -3, -4 and RECK mRNA transcript in HepG2 and MHCC97L cells. **B** shows that genipin exposure is responsible for the increased secretion of TIMP-1 in HepG2 and MHCC97L cells. The cells were transfected with scr negative control siRNA or siRNA against TIMP-1. Then cells were treated with genipin for 24H then medium was collected for ELISA determination of TIMP-1. *p<0.05,p<0.01 when compared with control; **C** shows that genipin up-regulates expression of TIMP-1. The cells were transfected with scr negative control siRNA or siRNA against TIMP-1. Then cells were treated with genipin for 24H and cell lysates were analyzed with immunoblotting.

**Figure 4:**
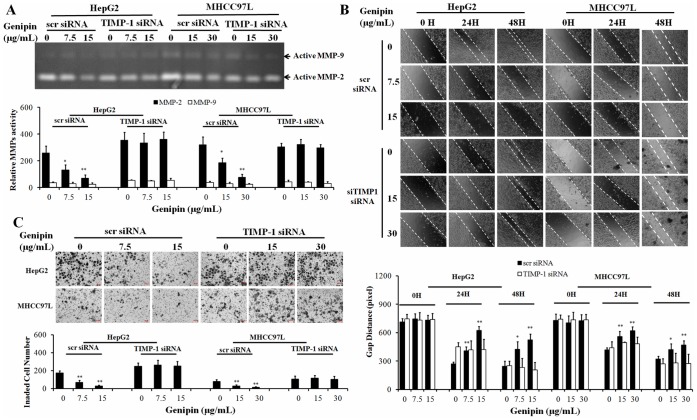
Upregulation of TIMP-1 expression is responsible for the MMP-2 inhibition by genipin in HCC cells. **A** shows that inhibition of TIMP-1 expression by RNA interference abolishes inhibition of MMP-2 activity by genipin. Cells were transfected with either scr siRNA or siRNA against TIMP-1 followed by genipin treated for 24H. The suppression of TIMP-1 by siRNA was verified as Fig. 3C shows. Medium was then collected for gelatin zymography analysis. Intensity of the band was quantified. *p<0.05, **p<0.01 when compared with control; **B** shows that inhibition of TIMP-1 attenuates genipin-suppressed HCC cell motility. Distance between the gap was quantified. *p<0.05, **p<0.01 when compared with control; **C** shows that inhibition of TIMP-1 expression attenuates HCC cell invasiveness suppressed by genipin. The invaded cell number was quantified. *p<0.05, **p<0.01 when compared with control.

We next determined the expression of endogenous inhibitors of MMP-2 activity, the TIMPs family and RECK, which have been reported to suppress MMP-2 activity in pathological conditions [14]–[18]. We found that the mRNA transcription of TIMP-1 was induced genipin intervention, while expression of other TIMPs and RECK remains unchanged (Fig. 3A). To further determine this, we measured the endogenous TIMP-1 level by western blot and secreted TIMP-1 in conditioned medium by ELISA assay. Increased expression of TIMP-1 could be observed in cells with genipin treatment, which is negative correlation with the down-regualtion of MMP-2 activities (Fig. 3B&C). TIMP-1 was reported to inhibit both MMP-2/9 activities in line [19], therefore the inhibition of MMP-2 activities in human hepatocellular carcinoma cells exposed to genipin may be attributable to up-regulation of TIMP-1. This was confirmed by the observation that the MMP-2 activities was restored when the increases of TIMP-1 expression by genipin was blocked by RNA interference in HCC cells (Fig. 4A). Furthermore, it was found that the inhibition of genipin on HCC cell motility and invasiveness could be attenuated by suppression of TIMP-1 up-regulation (Fig. 4B&C). Our findings confirm that increased expression of TIMP-1 is responsible for the inhibitory effect of genipin on MMP- activities in HCC cells.

### p38 MAPK Activation is Responsible for the in vitro Inhibitory Action of Genipin on HCC Cells Migration and Invasion

**Figure 5:**
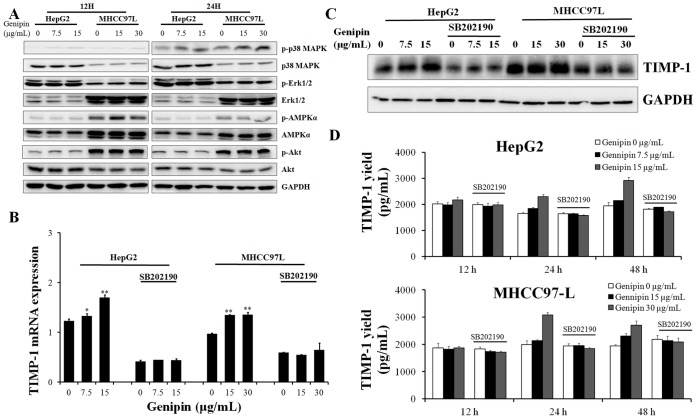
Activation of p38 MAPK signaling by genipin confers the overexpression of TIMP-1 in HCC cells. A shows that genipin activates p38 MAPK signaling in dose- and time- dependent manner. Cells were treated with genipin for 12 for 24H then protein was collected. The activation of p38 MAPK was analyzed by immunoblotting. **B** shows that inhibition of p38 MAPK repressed mRNA transcripts up-regulated by genipin treatment. Cells were treated with genipin for 12H in the presence or absence with SB202190 (20 µM) then total RNA was collected. The TIMP-1 mRNA transcripts were analyzed by qRT-PCR. *p<0.05, **p<0.01 when compared with control; **C** shows that inhibition of p38 MAPK suppressed the up-regulation of TIMP-1 protein expression induced by genipin. Cells were treated with genipin for 24H in the presence or absence with SB202190 (20 µM) then protein was collected for immunoblotting analysis. **D** shows that inhibition of p38 MAPK reduced TIMP-1 secretion. Cells were treated with genipin for 12, 24 or 48H in the presence or absence with SB202190 (20 µM) then medium was collected and TIMP-1 secretion was analyzed by ELISA assay. *p<0.05, **p<0.01 when compared with control.

**Figure 6:**
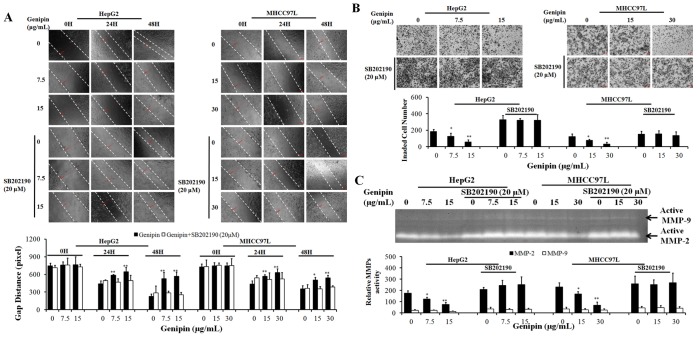
Inhibition of p38 MAPK attenuates genipin-suppressed HCC cell migration and invasion. A shows that inhibition of p38 MAPK signaling attenuates cell migration and invasion inhibition by genipin. Cells were pre-treated with p38 MAPK inhibitor, SB202190 (20 µM), for 30 min followed by being exposed to genipin. The cell migration was determined by wound healing assay. The distance between the gap was quantified. *p<0.05, **p<0.01 when compared with control. **B** shows that inhibition of p38 MAPK restores invasiveness of HCC cells. Cells were pre-treated with p38 MAPK inhibitor, SB202190 (20 µM), for 30 min followed by being exposed to genipin. The cell invasion through ECM was determined by chamber assay. Invaded cell number was counted. *p<0.05, **p<0.01 when compared with control. **C** shows that MMP-2 activity inhibition in HCC cells exposed to genipin was attenuated in the presence of SB202190. Cells were pre-treated with SB202190 (20 µM) for 30 min followed by genipin exposure for 24H. The MMP-2/9 activity in conditioned medium was determined by gelatin zymography.

Dose- and time-dependent manner of activation of p38 MAPK pathways by genipin treatment was observed in human hepatocellular carcinoma cells HepG2 and MHCC97L (Fig. 5A). 12H to 24H exposure of genipin was sufficient to activate p38 MAPK in HCC cells without altering the Erk1/2, AMPK, or Akt pathway activity. To further confirm the role of p38 MAPK activation in the anti-metastasis effect of genipin, we examined the function of p38 MAP in activating TIMP-1 expression. The presence of SB202190, the p38 MAP inhibitor could down-regulate the mRNA transcripts of TIMP-1 (Fig. 5B) as well as the TIMP-1 protein expression (Fig. 5C). Furthermore, Increased expression of TIMP-1 in HCC cells treated with genipin was suppressed in the presence of SB202190 (Fig. 5D). To further decipher the role of p38 MAPK pathway in the genipin’s inhibitory effect on cancer cell migration and invasion, we used SB202910, the p38 MAPK inhibitor, to block the activation of the p38 MAPK by genipin. We found p38 MAPK inhibition by SB202190 could significantly attenuate the inhibitory action of genipin on in vitro migration and invasion of HepG2 and MHCC97L cells (Fig. 6A&6B).As well, the suppression of MMP-2/9 activities by genipin in HCC cells was attenuated by co-treatment with p38 MAPK inhibitor SB202190 (Fig. 6C). These results indicate that activation of p38 MAPK pathway by genipin may suppress migration and invasion of HCC cells via increasing the expression of TIMP-1. Our result indicates that p38 MAPK activation is responsible for the in vitro inhibitory action of genipin on the migration and invasion of human hepatocellular carcinoma cells HepG2 and MHCC97L.

### Genipin Suppresses the Invasiveness of Tumor Cells in Orthotopicallly Implanted HCC Model

**Figure 7:**
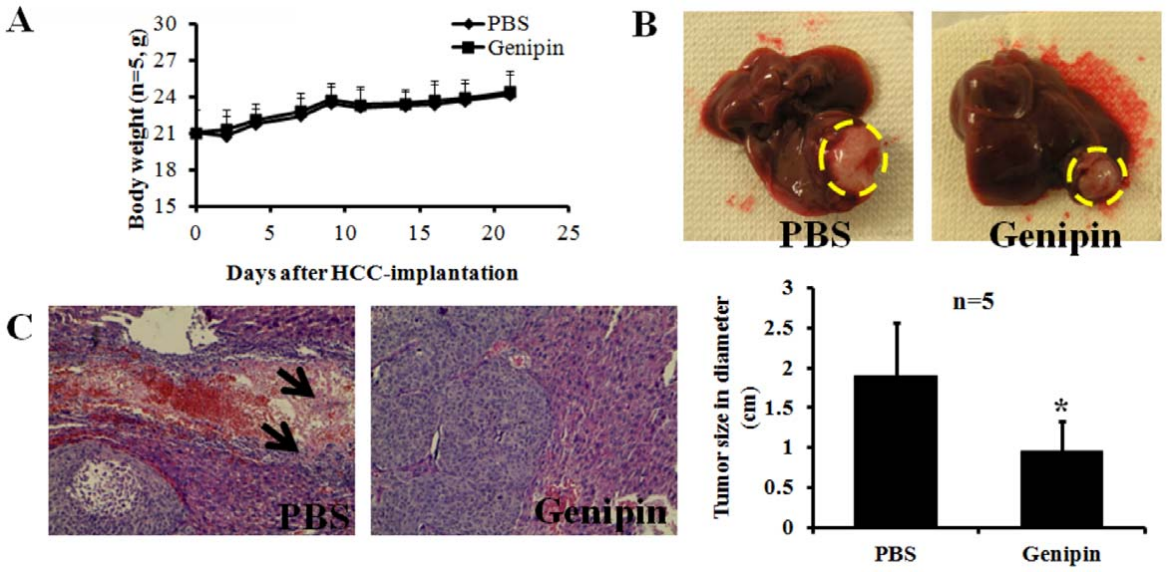
Genipin exhibits anti-invasive effect in orthotopically HCC-implanted mice. A shows Genipin has no toxic effect on the orthotopically HCC implanted model. MHCC97L cells were subcutaneously injected into the right flank of the nude mice to allow xenografted tumor growth. Once the solid tumor reaches about 1 cm in diameter, it was dissected out and cut into 1–2 mm small cubes, which was then implanted to the left liver lobe of the nude mice to allow orthotopical growth of HCC. Seven days later, the nude mice was randomized into two groups (n = 5). Treatment was conducted seven days after implantation (genipin 30 mg/kg/2 days, i.p.) and lasted for 3 weeks, while control group received same volume of PBS. Body weight of the mice was examined once per two days. No significant loss in body weight during treatment could be observed, indicating genipin may not exhibit potent toxicity to the mice. **B** shows that genipin treatment could reduce the tumor size in HCC-implanted mice. **C** shows that genipin suppresses tumor cell invasion to the normal tissue in orthotopically HCC implanted model. The liver of mice was dissected out and histological analysis was conducted with H & E staining. Significantly reduction of intrahepatic invasion of MHCC97L could be observed in genipin-treated mice.

The anti-metastatic potential of genipin in HCC was further examined with the orthotopically HCC-implanted mice. Mice were treated with either vehicle or genipin (30 mg/kg/2 days, i.p.) for 3 weeks. To examine the possible toxicity of genipin on the animal, we determined the body weight once per 2 days. No significant reduction of body weight was observed in genipin-treated mice, indicating that genipin may not present potent toxic action to the animal (Fig. 7A). Liver was dissected out at the end of the experiment, and the results showed that genipin could suppress the tumor size in HCC-implanted mice (Fig. 7B). Intrahepatic invasion of HCC cells to the normal tissue was examined by H&E staining. It was observed that in control group, tumor cells exhibit active metastasis effect, and venous infiltrate invasion could be found in mice treated with vehicle. Tumor cells are able to invade to blood vessel (the arrow indicated) in control group, while in genipin-treated mice, no potent invasion of tumor cell to the blood vessel could be observed. Absence of intrahepatic metastasis of tumor cell to the normal tissue could be found in genipin-treated mice (Fig. 7C).

## Discussion

**Figure 8:**
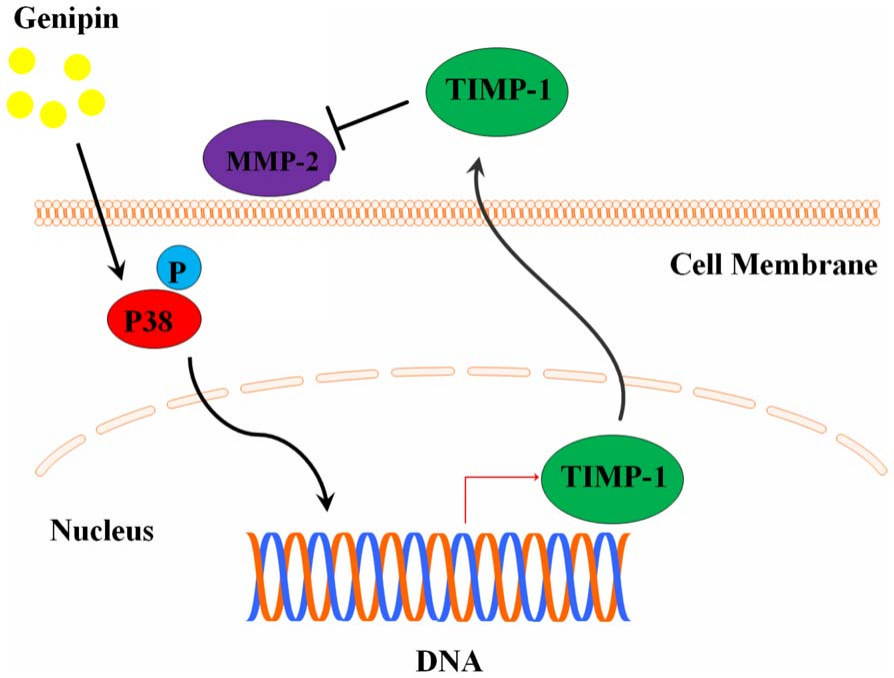
Overall scheme of the regulatory network involved in the inhibitory effect of genipin on HCC invasion.

Previous studies have reported some anti-migration and anti-invasion effect of genipin. Studies showed that genipin could suppress the alpha-TN4 lens epithelial cells and subconjunctival fibroblast migration induced by TGF-β [20], [21]. Genipin was shown to inhibit breast cancer cell MDA-MB-231 migration and to change the invasive phenotype of breast cancer cells [22] and UCP2 may be the possible target involved [23]. In this study, we reported for the first the anti-migration and anti-invasion effect of genipin in hepatocellular carcinoma. Genipin exhibits no significant toxicity to HCC cells HepG2 and MHCC97L, however, potent inhibition on cell motility and invasiveness through extracellular matrix could be found in HCC cells treated with genipin. Moreover, genipin was shown to suppress intrahepatic invasion of HCC cells in orthotopically implanted HCC animal model. Mechanism study exhibits that genipin is capable of down-regulating the gelatinase MMP-2 activities without suppressing their expression; up-regulation of TIMP-1, the endogenous inhibitor of MMP-2, may be responsible for the inhibition of MMP-2 activity. Although TIMP-1 should be responsible for the MMP-9 activity, the up-regulation of TIMP-1 could not exhibit statistically significance of MMP-9 inhibition in HCC cells with treatment of genipin. This may be due to the relative low-expression of basal level of MMP-9. Activation of p38 MAPK signaling was observed in HCC cells exposed to genipin, and inhibition of p38 MAPK signaling by SB202190 abolishes up-regulation of TIMP-1 by genipin, as well as attenuates MMP-2 inhibition by genipin. Systematic scheme of regulatory network of genipin in HCC was shown in Fig. 8. Our result indicates the possible potential of genipin as an adjuvant therapy in hepatocellular carcinoma.

The MMPs family is a group of enzymes involving in the tumor cell migration, invasion and metastasis by degeneration of extracellular matrix proteins such as collagens and proteoglycans [24]. Amongst the MMPs, gelatinases MMP-2/9 show dominant role in cancer progression [25], indicating the potential of MMP-2/9 as therapeutic target in the prevention of cancer metastasis. In our study, we found for the first time genipin is a specific inhibitor to MMP-2 activities in human hepatocellular carcinoma cells. Genipin has no effect on the expression level of MMP-2, indicating that genipin may possibly inhibit MMP-2 by directly being their inhibitor or by up-regulating their endogenous inhibitors. Direct exposure of human MMP-2 recombinant proteins to genipin has no effect on their activities, showing that genipin may be not act as a small molecule that can inactivate MMP-2. Instead, we found that the inhibitory effect of genipin on MMP-2 acts through up-regulating their endogenous inhibitor TIMP-1. Up-regulation of TIMP-1 was shown to suppress the tumor invasion and metastasis in various human cancers, which may be correlated with its inhibition on MMP-2/9 [26]–[28]. Although we could observe dose-dependent suppression of MMP-9 activity, there’s no significance between non-treated and treated cells statistically. Furthermore, since the basal levels of MMP-2 were dominantly and significant suppression of genipin on MMP-2 activity could be found in HCC cells, we concluded that genipin may target on MMP-2 majorly, which contributes to the anti-metastatic effect of genipin in hepatocellular carcinoma. Our results revealed that silencing the expression of TIMP-1 by RNA interference could significantly recover the activities of MMP-2 in genipin-treated HCC cells, as well as the motility and invasiveness of HCC cells. This exhibits that up-regulation of TIMP-1 is responsible for the anti-metastatic potential of genipin in hepatocellular carcinoma.

The p38 MAPK signaling is a stress-activated signaling pathway which responses when cells are receiving stress or cytokines. The recent studies revealed the role of p38 MAPK as a suppressor in tumor progression, which indicates its broader function other than stress response [29]. It was shown that knockout of p38αgenerates tumors in nude mice, presenting the unsuspended role of p38 MAPK in tumorigenesis [30]. The kinase activity of p38 was also demonstrated by an observation of slower tumor proliferation and metastasis in cancer cells with sustained activated p38 MAPK [31]. Furthermore, less active p38 MAPK was found in human hepatocellular carcinoma tissue in compared with normal liver, and the less active p38 was observed in tumor with larger size. These observations indicate both expression and activity of p38 MAPK are suppressed in human cancer, where the inhibition of p38 MAPK activity may be dominant in controlling tumor progression [32]. In our study, we found that genipin could potently activate p38 MAPK signaling at non-toxic dosages, which may be responsible for the anti-invasion and anti-metastasis effect of genipin in human HCC. The total level of p38 MAPK could not be over-expressed by genipin, which indicates that genipin may regulate the metastatic potential of HCC cell majorly through activating its p38 activity. Inhibition of p38 activity by SB202190 significantly recovered the invasiveness and metastatic potential of HCC cells exposed to genipin, indicating the central role of p38 activation in genipin’s anti-metastatic effect. Activators of p38 MAPK signaling were previously reported for their anti-metastatic potentials in human cancers. Quercetin was reported to inhibit the uPA signaling, which was related to increased p38 activity by quercetin in prostate cancer [33]. Celastrol activated-p38 MAPK was responsible for its inhibition on beta1 integrin function, cell-ECM adhesion, and phosphorylation of FAK [34]. Some previous studies also found TIMP-1 up-regulation by activated p38 MAPK in human cancer cells treated with chemotherapeutic agents cannabidiol and cisplatin [35], [36], which exhibit potent inhibition on human cancer cell invasion in vitro. Our results demonstrate that genipin presents similar anti-metastatic effect by activating p38 MAPK to induce TIMP-1 expression in hepatocellular carcinoma, indicating that genipin may be on the list of potential therapeutic agents against human cancer.

## Materials and Methods

### Chemicals, Antibodies and Reagent

Genipin, gelatin and 3-(4,5-Dimethylthiazol-2-yl)-2,5-diphenyltetrazolium bromide (MTT) were purchased from Sigma-Aldrich (USA). Rabbit monoclonal antibodies against MMP-2, MMP-9, E-cadherin, integrin β4, N-cadherin and TIMP-1 were purchased from Cell Signaling Techonolgies (USA). Rabbit polyclonal antibody against uPA was purchased from Santa Cruz (USA).

### Cell Line and Cell Culture

Human hepatocellular carcinoma cell line HepG2 was purchased from ATCC (USA); Human hepatocellular carcinoma cell line MHCC97L was kindly gifted by Dr. Man Kwan Department of Surgery, The University of Hong Kong, and has been used in our previous published works [4], [37]. Cells were maintained in DMEM medium with 10% Fetal Bovine Serum (Life Technologies, USA) and 1% penicillin/streptomycin (Life Technologies, USA) and incubated in a humidified atmosphere containing 5% CO_2_ at 37%.

### Cell Viability Assay

The cell viability after Genipin treatment was determined by MTT assay. Briefly, cells were seeded in 96-well plate and treated with series concentration of Genipin (0, 3.75, 7.5, 15, 30, 60 and 120 µg/mL) and incubated 24 and 48 h. All experiments were performed parallel with controls (0.1% DMSO). After incubation, 10 µl of 3-(4,5-Dimethylthiazol-2-yl)-2,5-diphenyltetrazolium bromide (MTT, 5 mg/ml, Sigma, USA) were added to each well and incubated at 37°C for 4 h. Then medium was removed and 100 µl of DMSO was added to each well. The absorbance of formazan formed was measured at 595 nm by Multiskan MS microplate reader (Labsystems, Finland).

### Wound Healing Assay

Cells were cultured in 24-well plate with 100% confluence. A gap was scrapped using a micro-pipette tip on the cell monolayer. Medium was removed and the monolayer was washed with warm PBS for 3 times. Then growth medium containing vehicle or different concentrations of genipin was added to each well and graphics were obtained after 0, 24 and 48 h incubation.

### Matrigel Matrix Invasion Assay

The invasion chamber assay was conducted with Millicell Cell Culture Insert (24-well PCF 8.0 µl, Millipore). Briefly, the inserts standing in 24-well cell culture plate were coated with 100 µl 1 mg/mL Matrigel Matrix (BD Falcon, USA) in serum-free DMEM medium and air-dried. 100 µl serum-free medium containing 5×10^4^ cells were added to the insert.0.5 ml of DMEM medium containing 10% FBS and indicated concentrations of genipin was added to each well of the 24-well plate. This follows incubation in 5% CO_2_ at 37% for 48H. Then the non-invading cells on the upper surface of Matrigel matrix were removed by cotton swabs. The cells that invaded across the collagen to the lower surface of the membrane were fixed by ice cool 100% ethanol and stained by 2% Crystal Violet (Sigma Aldrich, USA). Photographs of the stained invaded cells (5 random fields/culture) were taken under an inverted microscope at 200x and the mean number of cells of the 5 fields was recorded. The data were then expressed as the average number (±SD) of cells from 5 fields that invaded from each of 3 experiments performed.

### Gelatin Zymography

The gelatin zymography was performed to determine the activity of MMP-2 and MMP-9. Briefly, medium was collected and mixed with 3X loading buffer. Protein in medium was then separated in 10% SDS-page gel containing 1 mg/ml gelatin. After running, the gel was incubated in the 2.5% triton-X in deionized water for renaturing with gentle agitation for 30 minutes at room temperature. Then the gel was incubated in developing buffer (50 mM Tris-HCl, 0.2 M NaCl, 5 mM CaCl_2_, 0.02% Brij35) overnight with gentle shaking. The gel was stained Coomassie Blue R-250 for 30 minutes and then washed. The gel was visualized under a chemiluminescence imaging system (Biorad, USA) and Figures were captured.

### In vitro MMP-2/9 Activity Assay

The MMP-2/9 activity dynamics were evaluated using MMP-2/MMP-9 colormetric drug discovery kits (Enzo, USA) under the manufacturer’s instruction. In brief, the human recombinant MMP-2 or MMP-9 was mixed with 30 µg/mL genipin or NNGH 100 nM inhibitor as negative control. The mixture was incubated at 37°C for 45 min then substrate was added. The plate was immediately read at 405 nm by Multiskan MS microplate reader (Labsystems, Finland). Continuous recording was made at 1 min interval for 10 min. The dynamic changes of MMP-2/9 were determined by the slope of line fit to liner portion. The experiment was performed in duplicate.

### TIMP-1 ELISA Assay

The TIMP-1 expression was determined by human TIMP ELISA kit (ExCell Biology, Shanghai, China) under the manufacturer’s instruction. Briefly, samples were added to the microwell plate with human TIMP-1 monoclonal antibody coating in combination with biotinylated antibody and incubates for 2 h at room temperature. Equal volume of assay diluents buffer was added to one well as empty control. All experiments were conducted in triplicate to avoid handling error. Then wells were washed six times and the HRP-conjugated streptavidin was added to each well except the empty control for 1 h incubation at room temperature. Then plate was washed as described above and TMB substrate solution was added for 30 min incubation in dark at room temperature. At the end of incubation, 100 µl stop solution was added to each well and the absorbance at 450 nm was read with reference at 630 nm using Multiskan MS microplate reader (Labsystems, Finland).

### Quantitative Real-time PCR

Total RNA of cells with or without berberine intervention was extracted and purified using RNeasy Mini Kit (Qiagen, Germany) following the manufacturer’s instruction. RNA samples were subject to QuantiTech Reverse Transcription Kit (QIAGEN, Germany) for the reverse transcription. The quantitative real-time PCR (qRT-PCR) was conducted by LightCycler 480 SYBR Green I master (Roche, USA) with 1 µM primers (Table 1) LightCycler 480 real-time PCR system (Roche, USA). The expression of β-actin was used as endogenous control for the normalization of gene expression.

**Table 1:** Primers used for real-time quantitative PCR.

gene symbol		Sequence (5′ to 3′)
*ACTIN*	FWD	CCAACCGCGAGAAGATGA
	REV	CCAGAGGCGTACAGGGATAG
*TIMP-1*	FWD	GGGCTTCACCAAGACCTACA
	REV	TGCAGGGGATGGATAAACA
*TIMP-2*	FWD	GAAGAGCCTGAACCACAGGT
	REV	CGGGGAGGAGATGTAGCAC
*TIMP-3*	FWD	TGCAACTCCGACATCGTG
	REV	AAGGGCCCCTCCTTTACC
*TIMP-4*	FWD	CAGACCCTGCTGACACTGAA
	REV	CTCCAGAGGCACTCGTTAGG
*RECK*	FWD	CAAGTGTCCTTCGCTCTTGG
	REV	CACATAATGGGCAACAAGCA
*MMP-2*	FWD	CCCCAAAACGGACAAAGAC
	REV	CTTCAGCACAAACAGGTTGC
*MMP-9*	FWD	GAACCAATCTCACCGACAGG
	REV	GCCACCCGAGTGTAACCATA

### Immunoblotting

Cells was harvested using a micro-scrapper (Corning, USA) and then lysed with RIPA buffer supplemented with proteinase inhibitor (1% PMSF, 0.5% apotinin and 0.5% leupitine) and phosphatase inhibitor (1 mM Na_3_VO_4_ and 1 mM NaF) on ice for 30 minutes and then centrifuged at 14,000 rpm at 4°C for 25 minutes. The supernatant was transferred to a new tube and protein concentration was determined using BSA as standard. Equal amounts of protein were resolved by SDS-PAGE and transferred onto a polyvinylidene fluoride membrane (PVDF, Biorad). Then the membrane was blocked with 5% BSA overnight at 4°C. The membrane was then incubated with primary antibodies at 4°C overnight followed by incubation with appropriate secondary antibodies (Abcam, UK) at room temperature for 2 hours. The immunoreactivites were detected using ECL Adanced kit (GE Healthcare, UK) and visualized using a chemiluminenescence imaging system (Biorad, USA).

### Evaluation of Anti-metastatic Potential of Genipin in vivo

The anti-metastatic potential of genipin was evaluated with orthotopically HCC-implantation animal model, which has been described in our previous study [37]. In brief, approximately 1×10^7^ MHCC97L in 0.2 mL was subcutaneously injected into the right flank of the male nude mice to allow solid tumor growth. Once the tumor researched about 1 cm in diameter, the mice was sacrificed by injecting i.p. overdose of Phenobarbital (200 mg/kg) and the tumor was removed and cut into 1–2 mm small cubes, which were then implanted into the left liver lobe of the male nude mice, which were anaesthetized by etamine/xylazine (67 mg/kg/6 mg/kg, i.p.). To minimize analgesics after animal surgery, mice were received meloxicam (1.5 mg/kg) orally for a week. Seven days later, the nude mice was randomized into two groups (n = 5). One group received genipin 30 mg/kg/2 days, i.p. for 3 weeks and the other received same volume of PBS as control. At the end of experiment, the mice were sacrificed by injecting overdose of Phenobarbital (200 mg/kg) and the liver was dissected out. The tumor size was calculated. The liver was then cut and subject to H & E staining to evaluate the intrahepatic invasion of MHCC97L cells (40× magnification in light microscope). All protocols in animal handling complied with the guidelines of the Laboratory Animal Centre of the University of Hong Kong. Animals were processed (including drug treatment and sacrifice) in accordance with the international guidelines for laboratory animals. The animal experimental protocol was approved by the Committee on the Use of Live Animal in Teaching and Research (CULATR) in The University of Hong Kong.

### Statistical Analysis

Results were analyzed using student T-test and expressed as mean ± SD.

### Conclusions

In conclusion, we reported the anti-metastatic potential of genipin in human hepatocellular carcinoma. Genipin exhibits no potent cytotoxicity to hepatocellular carcinoma cell HepG2 and MHCC97L and no-toxic doses of genipin could significantly suppress HepG2 and MHCC97L cell migration as well as invasion through extracellular matrix. Genipin is capable of inhibiting MMP-2 activity without alteration their mRNA and protein expression in HCC cells, which may play a key role in its anti-metastatic effect. Up-regulation of TIMP-1, the endogenous inhibitor of MMP-2 in HCC cells with treatment of genipin, could be observed and abolishment of TIMP-1 expression by RNA interfering attenuates anti-invasive action of genipin. Activation of p38 MAPK signaling could be observed in genipin-treated HCC cells, which is responsible for the overexpression of TIMP-1 and inhibition of cell migration and invasion. Orthotopically HCC-implantation model demonstrates that genipin could suppress intrahepatic invasion of HCC cells into the normal tissue. Taken together, our results demonstrate the anti-metastatic potential of genipin in hepatocellular carcinoma, and the MMP-2 inactivation by p38-driven TIMP-1 overexpression may be involved as the major mechanism in genipin’s anti-metastatic effect.
